# Paeonol Induces Protective Autophagy in Retinal Photoreceptor Cells

**DOI:** 10.3389/fphar.2021.667959

**Published:** 2021-05-26

**Authors:** Daowei Zhang, Jiawen Wu, Jihong Wu, Shenghai Zhang

**Affiliations:** ^1^ Eye Institute, Eye and ENT Hospital, College of Medicine, Fudan University, Shanghai, China; ^2^ Shanghai Key Laboratory of Visual Impairment and Restoration, Science and Technology Commission of Shanghai Municipality, Shanghai, China; ^3^ State Key Laboratory of Medical Neurobiology, Institutes of Brain Science and Collaborative Innovation Center for Brain Science, Shanghai, China; ^4^ Key Laboratory of Myopia, Ministry of Health, Shanghai, China

**Keywords:** paeonol, retinal photoreceptor, apoptosis, oxidative stress, autophagy

## Abstract

**Background:** Retinal photoreceptor (RP) cells are widely involved in retina-related diseases, and oxidative stress plays a critical role in retinal secondary damage. Herein, we investigated the effectiveness and potential mechanisms of autophagy of paeonol (Pae) in terms of oxidation resistance.

**Methods:** The animal model was induced by light damage (LD) *in vivo*, whereas the *in vitro* model was established by H_2_O_2_ stimulation. The effectiveness of Pae was evaluated by hematoxylin and eosin, terminal deoxynucleotidyl transferase dUTP nick end labeling assay, immunofluorescence, transmission electron microscopy, electroretinogram, and Western blot analysis *in vivo*, and the underlying mechanisms of Pae were assessed by Cell Counting Kit-8 assay, reactive oxygen species (ROS) assay, and Western blot analysis in 661W cells. We mainly evaluated the effects of Pae on apoptosis and autophagy.

**Results:** Increased apoptosis of the LD-induced and decreased autophagy of RPs were mitigated by Pae treatment. Pea, which increased the expression of mitochondrial functional protein cytochrome c, reversed the decreased cell viability and autophagy induced by oxidative stress in 661W cells. Experiments showed that autophagy was downregulated in PINK1/Parkin dependent and the BNIP3L/Nix dependent pathways under H_2_O_2_ stimulation and was upregulated by Pae treatment. Pae increased the cell viability and reduced ROS levels through autophagy.

**Conclusion:** Pretreatment with Pae preserved RP cells by enhancing autophagy, which protected retinal function.

## Introduction

Retinal photoreceptor cells (RPs) are essential in the process of normal visual transduction and are associated with a broad variety of vision-threating diseases such as glaucoma, age-related macular degeneration, retinopathy of prematurity and so on. The injury of RPs could be mostly attributed to constant exposure to highly oxidative environment owing to high metabolic activity, large consumption of oxygen and photochemical damage from excess light in retina (Sundermeier and Palczewski, 2016). The imbalance between elevated oxidative stress and antioxidant defense mechanisms could cause dysfunction of mitochondria and other intracellular organelles in RPs (Lefevere et al., 2017) and even trigger unreversible cell death, which highlight the importance of developing therapeutic interventions to attenuate oxidative damage and protect RPs.

Paeonol (Pae; 2′-hydroxy-4′-methoxyacetophenone) is a major phenolic acid compound derived from the root bark of the Moutan Cortex and serves as a natural active ingredient in Chinese herbal medicines (Choy et al., 2018). It has been found to possess pharmacological effects including sedation, analgesia and immunoregulatory, and exert anti-tumor (Ramachandhiran et al., 2019) and anti-inflammatory response (Chen et al., 2012). Zhao et al. reported that Pae exhibited neuroprotective effect in a subacute/chronic cerebral ischemia rat model by effectively alleviating neurological impairment and neuronal loss (Cai et al., 2014). Zhou et al. also found that Paeonol intervention slowed down the pathogenic processes in a rat model of Alzheimer’s disease (Zhou et al., 2011), suggesting a potential anti-oxidant effect of Pae. Another study observed therapeutic effects of Pae on Parkinson’s disease in mice by decreasing the damage from oxidative stress (Zhao et al., 2018). To the best of our knowledge, there have been no reports demonstrating the effects of Pae on RPs damage.

It is known that autophagy plays essential roles both in physiological processes, such as cell growth, cell differentiation and cell death, and in pathophysiological processes, including the adaptation to oxidative stress and the maintenance of cell homeostasis. Autophagy is a cellular degradation and recycling process which provides raw materials for the reconstruction of intracellular components by recycling dysfunctional organelles and misfolded proteins. Moreover, mitochondrial related autophagy, which refers to the selective removal of mitochondria by autophagy, is of increasing interest to researchers, as mitochondrial dysfunction is closely related to various retinal diseases. Prior studies have proposed that autophagy participated in cytoprotective response to damage of RPs (Shi et al., 2016), and autophagy was also highly enriched in RPs (Boya et al., 2016). PINK1/Parkin, Nix/BNIP3L and FUNDC1 are three main signal pathways involved in the process of autophagy, with Nix and FUNDC1 promoting autophagy in response to hypoxia (Li et al., 2018; McWilliams et al., 2019), and PINK1/Parkin mediating autophagy to cope with oxidative stress and other non-hypoxic stressors. Besides, Beclin-1 is involved in the control of autophagy by regulating the initiation and nucleation phases of autophagosome formation and the process of phagocytosis and endocytic trafficking.

This study sought to answer the following specific research questions: whether Pae possesses anti-oxidant effect and neuroprotective effect on RPs both *in vivo* and *in vitro* and whether the underlying mechanisms are related to autophagy in RPs. The study could provide a potential approach to protect RPs.

## Materials and Methods

### Animal Experiments Design

Animal experiments were conducted in accordance with the Guide for the Care and Use of Laboratory Animals published by the US National Institutes of Health (NIH Publication No. 85-23, revised 2011) and the guidelines on the ethical use of animals of Fudan University. Adult male Sprague–Dawley (SD) rats weighing approximately 180 g (SLAC Laboratory Animal Co., Ltd., Shanghai, China) were used in the experiment. The rats were housed with a daily 12-h light/12-h dark cycle and had free access to food and water. A total of 45 SD rats were randomized into the normal control (NC) group (*n* = 10), the light damage (LD) group (*n* = 10), the LD + vehicle group (*n* = 10, daily intraperitoneal injection of 1 μl DMSO), and the LD + Pae group (*n* = 15, daily intraperitoneal injection of 1 μl Pae, 80 mg/kg). After one week of Pae intraperitoneal injection, the electroretinogram (ERG) function was detected 3 days after 24-h blue light exposure (wavelength of 400–440 nm). The rats were then sacrificed with excessive abdominal anesthesia of chloral hydrate (600 mg/kg), and eyeballs were harvested for either immediate or future use.

### Hematoxylin and Eosin Staining

The eyeballs from each group were harvested, enucleated and immediately fixed in 4% paraformaldehyde for 24 h. Then the samples were embedded with paraffin, serially sectioned (5-μm/section) and stained with hematoxylin and eosin (Takara: C0105S). The sections were observed using a light microscope (Leica, Wetzlar, Germany), and the slices cutting cross-sectionally through the eyeball and the optic nerve were selected. The images photographed were all location matched, and the thickness of different retinal layers were measured at the points located approximately 250 μm from the optic nerve. Each selected section was measured five times and averaged to obtain the values for one sample.

### Fluorescence Staining

4% Paraformaldehyde-fixed and optimal cutting temperature compound-embedded rat eyes of every groups were sectioned at a thickness of 10 μm and subjected to TUNEL assay (Takara: C1089) to detect apoptotic cells. TUNEL staining was performed in accordance with the manufacturer’s instructions (Zhu et al., 2010). After counterstaining with DAPI (1:2000; Life Technologies, Carlsbad, CA, United States) for 10 min, the sections were observed using a confocal microscope (Leica SP8, Hamburg, Germany).

### Electron Microscopy

A piece of posterior pole tissue (1 mm × 1 mm × 1 mm) of eyes from each group was rapidly dissected on ice after enucleation, and fixed via 2.5% glutaraldehyde in 0.1 M phosphate buffer at 4°C for 2–4 h. After washing in 0.1 M phosphate buffer for 3 times, the samples were post-fixed with 1% osmic acid at 4°C for 2 h and washed again. The samples were then dehydrated using an ascending alcohol series, infiltrated with a 1:1 mixture of resin and acetone and embedded in epoxy resin. After being polymerized in a 60°C oven for 48 h, 60–80 nm ultrathin sections were obtained by using an ultramicrotome (Leica, Leica UC7). The sections were double stained with lead citrate and uranyl acetate (each for 15 min) and examined under a transmission electron microscope (TEM; HITACHI, HT7700).

### Measurement of Electroretinogram

The ERG was measured to evaluate the RP function of rats before intraperitoneal injection and three days after retinal light damages separately and was recorded by an Espion Diagnosys System (Diagnosys, Littleton, MA, United States). As described in Miyai et al. (2019), after 24 h of dark adaptation, the rats were given intraperitoneal anesthesia, and their pupils were dilated with phenylephrine hydrochloride and tropicamide (0.5%). Two wire loop electrodes were placed on the corneal surface of the eyes and served as the ERG signal-recording electrodes. In addition, two subdermal needle electrodes were inserted into the base of the tail and nasal part and separately served as the ground electrode and the common reference electrode. Retinal responses were recorded for 30 min.

Light stimulation was performed using a white LED following the protocol described in Miyai et al. (2019). Dark and light adaptation was performed in four steps, and the light intensity was switched from weak to strong. Electroretinographic waveforms were recorded and sampled, and the data were analyzed by using a Diagnosys digital acquisition system. The waveforms of ERG were measured from trough to peak (Lazarou et al., 2015), and the values of ERG amplitudes were compared among the four groups.

### Cell Culture

A photoreceptor cell line, 661W cells, was cultured in DMEM (Invitrogen: 11965092) containing 10% fetal bovine serum and 1% penicillin/streptomycin under normal condition (5% CO_2_, 37°C) as previously described (Birgisdottir et al., 2013). Cells of the passages 15–20 were used in the following experiments.

### Cell Counting Assay

The 661W cells were seeded in 96-well plates at a density of 1 × 10^4^ cells per well, and grouped as follows: control cells, cells exposed to H_2_O_2_, cells treated with Pae, and cells incubated with both H_2_O_2_ and Pae. The cells were treated with different concentrations of H_2_O_2_ (0–200 μM) and Pae (0–200 μM) for different time periods (0, 24, 48, 72, 96 h). After the cells were grown to approximately 80–90% confluency, Cell Counting Kit-8 (CCK-8) (Dojindo: CK04) assay was performed following the manufacturer’s protocol. The absorbance of each well was read at 450 nm by a microplate reader (BioTek, United States).

### Western Blot Analysis

Briefly, retinal tissues and cultured cells were lysed in radio-immunoprecipitation assay buffer (ASPEN, China) containing protease inhibitor cocktail (ROCHE). All sample extracts were electrophoresed by SDS-PAGE (ASPEN, China) and electrophoretically transferred to PVDF membranes. The membranes were then blocked with 5% skim milk at room temperature for 1 h, incubated with primary antibody diluent at 4°C overnight and incubated with horseradish peroxidase-coupled secondary antibodies. The blots were developed using enhanced chemiluminescence (ECL) and the signals were captured in a dark room. The immunoreactive bands were analyzed in triplicate by ImageJ, with GAPDH being used as a loading control.

Primary antibodies were as follows: anti-LC3 (1:1,000, Cell Signaling Technology/CST: 3868), anti-Bax (1:1,000, CST: 2774), anti-Bcl-2 (1:1,000, CST: 9942), anti-casepase-3 (1:1,000, CST: 9662), anti-p62 (1:1,000, CST: 8025), anti-Beclin-1 (1:2000, CST: 3738), anti-PINK1 (1:2000, CST: 6946), anti-Parkin (1:2000, CST: 4211), anti-Optineurin (1:2000, CST: 58981), anti-DNP52 (1:2000, CST: 60732), anti-BNIP3L/Nix (1:2000, CST: 12396), anti-BNIP (1:2000, CST: 44060), and anti-GAPDH (1:1,000, CST: 8884).

### Mito-Sox Assay

The 661W cells were seeded in 6-well plates at a density of 1 × 10^5^ cells per well and treated with H_2_O_2_ (100 μM) and/or Pae (50 μM) for 24 h after the cells were grown to approximately 80% confluency. Mitochondrial reactive oxygen species (ROS) were measured with a ROS Detection Kit (Takara: S0033S) according to the manufacturer’s instructions.

### Statistical Analyses

All experiments were repeated more than three times. Data were presented as mean ± standard deviation. One-way ANOVA and Bonferroni’s multiple-comparisons test were performed to compare the between-group difference. All statistical tests were performed with SPSS version 20 (IBM, United States) and *p* < 0.05 were considered statistically significant.

## Results

### Blue Light-Induced Retinal Photoreceptors Loss and Retinal Dysfunction Were Mitigated by Paeonol Treatment

**FIGURE 1:**
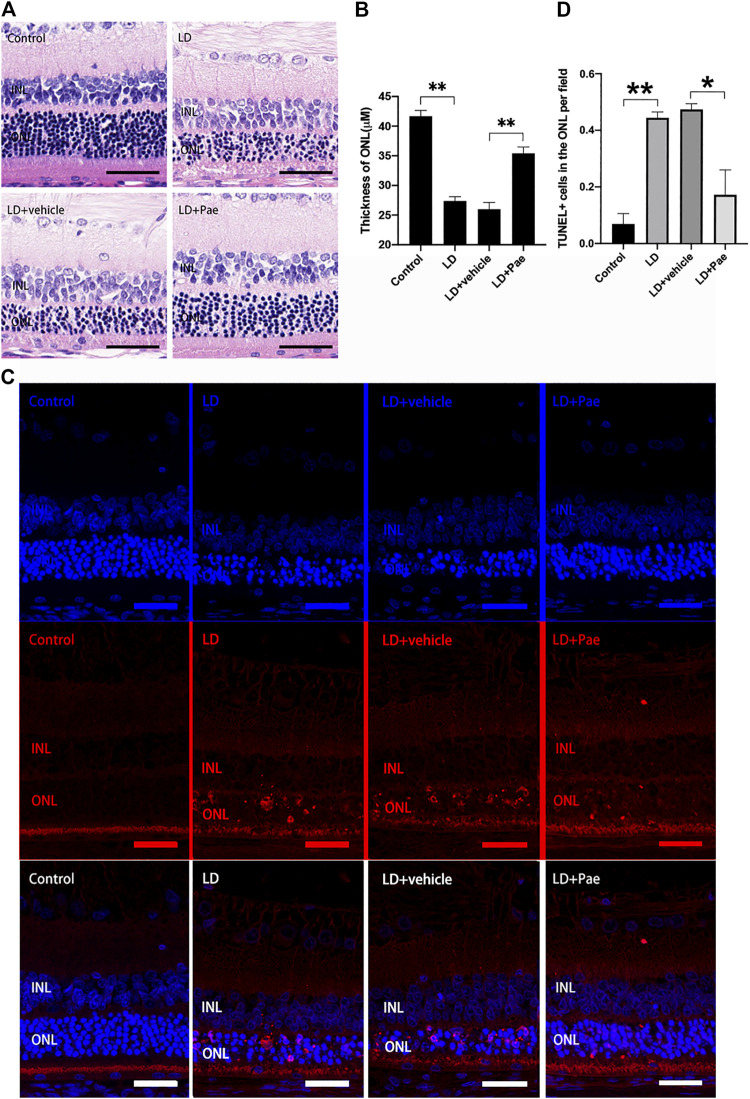
Pae mitigated light-induced RP loss. **(A)** HE images of representative retinal characterized morphological changes. Light exposure caused severe degeneration on retina, particularly of the ONL. Pae can reduce the damage, representing by a clearly thicker photoreceptor layer. **(B)** Statistical analysis of ONL thickness of each group. **(C)** Representative TUNEL-stained retinal sections showed the apoptotic cells three days after light damage. Light exposure caused a rapid increase of apoptotic cells, especially in the ONL. Less TUNEL-positive cells were visible after Pae treatment. **(D)** Statistical analysis of TUNEL^+^ cells of each group. Data show mean ± SD. (*n* = 6–8 for each group) **p < 0.01 *p < 0.05. Scale bar: 50 μm.

Exposure to blue light successfully induced the LD model in SD rats. Compared with the control group, significant thinned outer nuclear layers (ONL) were observed in the LD group and LD + vehicle group, whereas the thickness of ONL was effectively preserved after the administration of Pae (Figures 1A,B). It has been reported that apoptosis and autophagy appear to markedly accelerate three days after light injury, thus apoptosis in retina was examined by TUNEL assay three days after LD modeling. Apoptotic activity was almost absent for the control group, while an increasing number of TUNEL-positive cells were detected in the retina of the LD group and LD + vehicle group, especially located in ONL. Similarly, Paeonol treatment effectively attenuated light-induced RPs apoptosis in the LD + Pae group (Figure 1C).

**FIGURE 2:**
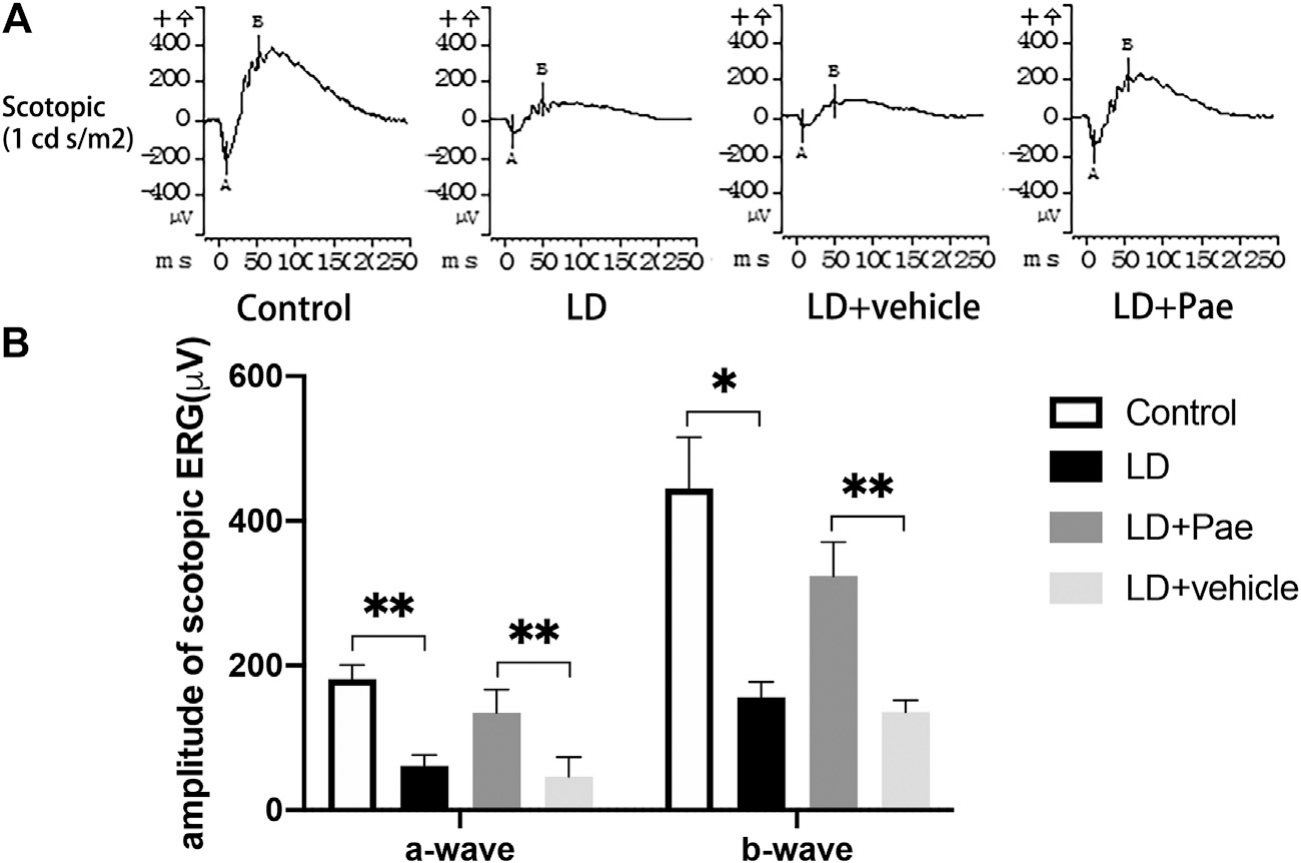
Pae protects RP function from LD. **(A)** Representative a- and b-waveforms of scotopic ERG. Light injury caused dysfunction of RP, showing by the drastically decreased amplitudes of a- and b-waves. Pae protected the RPs by sustaining the waveforms partly. **(B)** Statistical analysis of a- and b-waves of scotopic ERG. Data show mean ± SD. (*n* = 6–9 for each group) *p < 0.05, **p < 0.01.

The ERG reflects broad-scale retinal function, with the a- and b-waves indicating photoreceptor and second-order cell responses, respectively. As shown in Figure 2, the decreased amplitudes of a- and b-waves on scotopic ERG caused by LD were significantly mitigated by Paeonol treatment.

The above results indicated that Pae exerts protective effects on RPs by ameliorating morphological and functional damage induced by blue light exposure.

### Ultrastructural Features of Autophagy in the Retina of Light Damage Rats With and Without Paeonol Treatment

**FIGURE 3:**
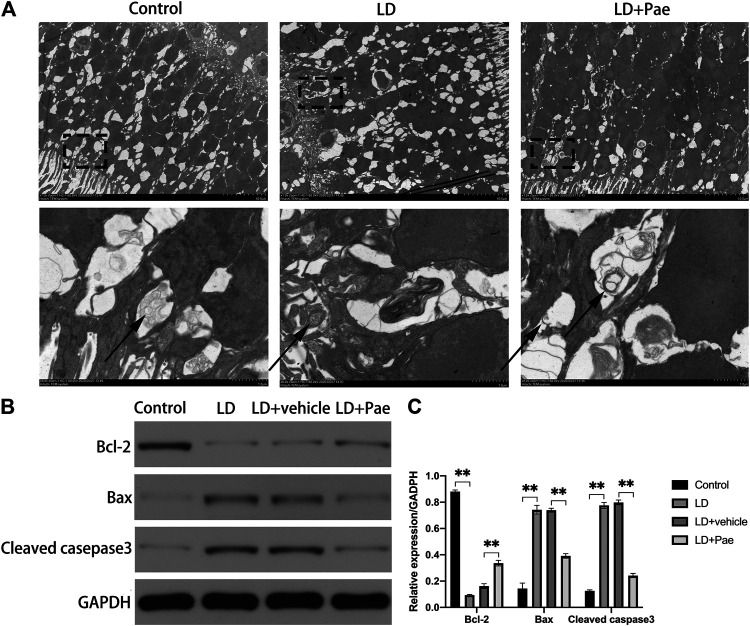
Effect of Pae on ultrastructural structure of autophagosome on RPs of each group and apoptosis of retina. **(A)** Ultrastructural structure of autophagosome formation of each group. **(B, C)** Expression of Bax and Cleaved casepase3 protein were decreased, while expression of Bcl-2 protein was upregulated three days after light damage. Autophagosomes were pointed by arrows. Data show mean ± SD. (*n* = 6–9 for each group) **p < 0.01.

The visualization of double-membrane compartments via TEM was considered as the gold standard for identifying autophagosomes (Pickford et al., 2008). As shown in Figure 3A, we found that cells from the NC group contained healthy mitochondria, which were easily recognizable in the normal cytoplasm. On the contrary, numerous double-membrane vacuoles accompanied by a few accumulated autophagic compartments were observed in cells of the retina subjected to 3 days after LD. Meanwhile, in Pae-treated retina, both newly formed and mature autophagosomes could be detected. Compared with increased autophagosome formation in the LD + Pae group, autophagy was inhibited in LD retina due to reduced autophagy induction, not by an increased autophagic flux.

### Light Damage-Induced Apoptosis in the Retina Was Attenuated by Paeonol Treatment

As shown in Figure 3B, Western blot analysis was used to evaluate the apoptosis in retina. Cleaved caspase-3 levels in the retina of the LD and LD + vehicle groups were remarkably higher than those in the NC group, which could be reversed by Pae treatment. Meanwhile, the Bax expression levels in the LD and LD + vehicle groups were remarkedly higher than those in the NC group. In addition, Bcl-2 levels were significantly reduced in retinas of the LD and LD + vehicle groups compared with those in the NC group. Pae significantly inhibited the up-regulation of Bax and down-regulation of Bcl-2. These results indicated that Pae might exert anti-apoptotic effects in the retina. This conclusion was consistent with the results of TUNEL assay (Figure 1C).

### Autophagy Was Up-Regulated by Paeonol Treatment in the Retina of Light Damage Rats

**FIGURE 4:**
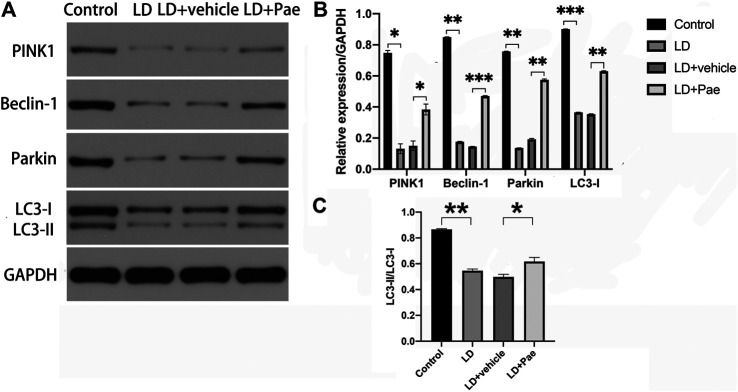
Impacts of Pae treatment on mitophagy in light-exposed RP. Western blot analysis of LC3-I and -II and Beclin-1 proteins in light injured RP **(A, B, C)** showed that in the LD and LD + vehicle groups, the expression levels of which were notably downregulated. Furthermore, the conversion rate from LC3-I to LC3-II in both groups was significantly decreased compared with that in the NC group, suggesting the absence of LC3-II and LC3-I. Pae treatment can largely reverse the damage. Western blot analysis of PINK1 and parkin in light injured RP **(A, B)** showed they were remarkably downregulated in the LD and LD + vehicle groups compared with the NC group, which can be significantly mitigated by Pae. The ratio of LC3-II to LC3-I was performing **(C)**. Data show mean ± SD. (*n* = 6–9 for each group) *p < 0.05, **p < 0.01, ***p < 0.001.

LC3-II and Beclin-1 are two major autophagy markers, and the PINK1/Parkin pathway is one of the important pathways in autophagy. The expression of LC3 and Beclin-1 in the retina was measured to evaluate the alteration of autophagy (Figure 4). In the LD and LD + vehicle groups, the expression levels of LC3-I and LC3-II were notably downregulated. In particular, the conversion rate from LC3-I to LC3-II in both groups was significantly decreased compared with that in the NC group, suggesting the absence of LC3-II and LC3-I. The ratio of LC3-II to LC3-I was higher in LD + Pae group comparing with it in LD + vehicle group. PINK1 and Parkin were remarkably downregulated in the LD and LD + vehicle groups compared with the NC group. Correspondingly, as the key regulatory protein for autophagy, Beclin-1 was also reduced. Moreover, the alterations of autophagy markers and autophagy pathway were significantly mitigated by Pae treatment. The above results indicated that LD damage in retina caused the resistance of autophagosome formation, the decreased autophagic flux and the block of autophagy, which fortunately Pae can improve to a much better extent.

### Decreased Cell Viability and Increased Apoptosis Induced by H_2_O_2_ Were Mitigated by Paeonol Treatment in 661W Cells

**FIGURE 5:**
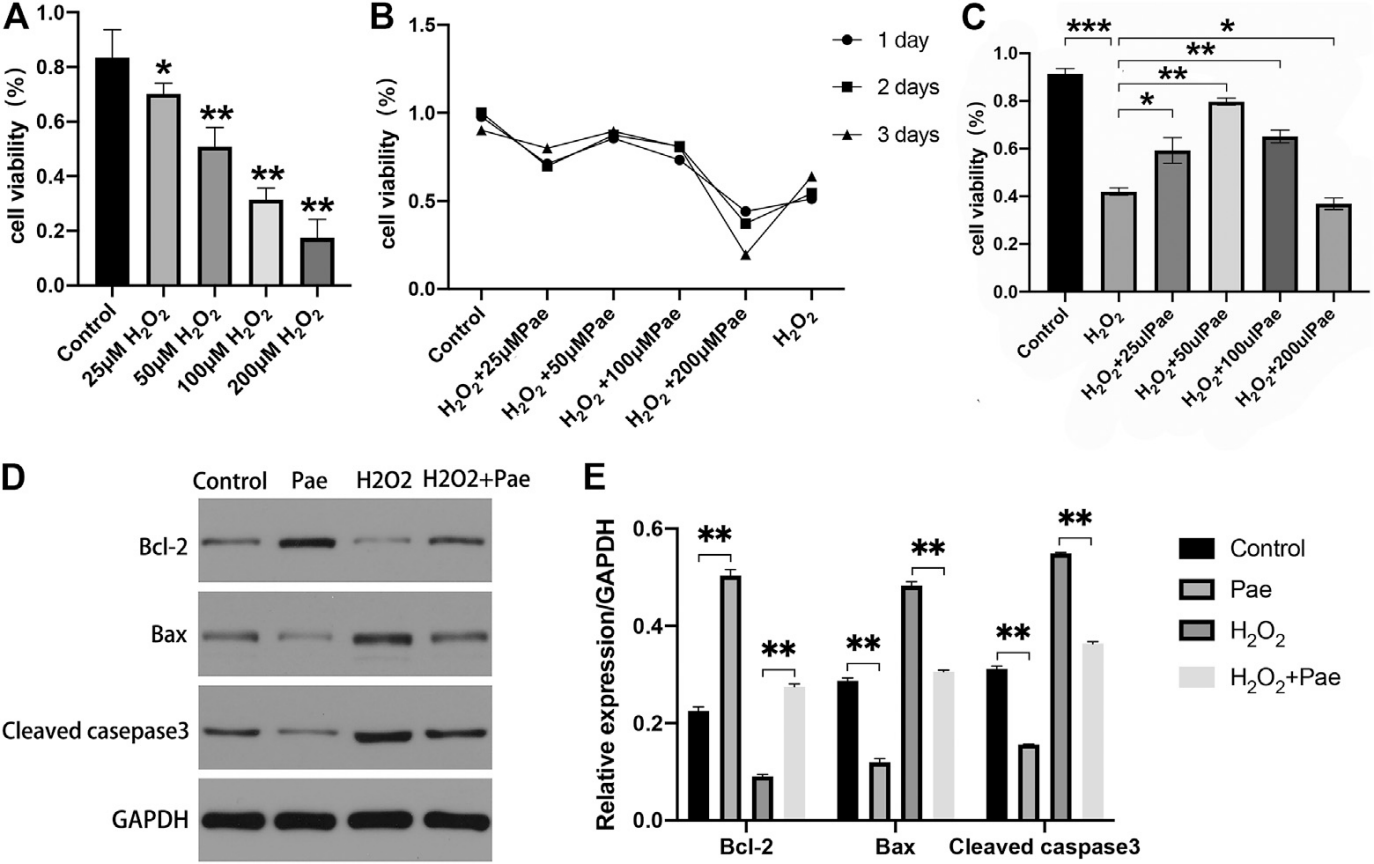
Effects of Pae on H_2_O_2_-induced decrease of viability and enhancement of apoptosis in 661W cells. **(A)** Cells were stimulated with 0, 25, 50, 100 or 200 μM H_2_O_2_, and cell viability was measured by CCK- 8 assay. Non-treated cells were acted as control. **(B)** Cells were stimulated by 100 μM H_2_O_2_ with 0, 25, 50, 100 or 200 μM Pae and Control group for 1, 2 or 3 days, and cell viability was measured by CCK- 8 assay. **(C)** Cells without treatment and were treated with 100 μM H_2_O_2_ alone or with the presence of 25, 50, 100 or 200 μM Pae, and cell viability was measured by CCK- 8 assay. **(D, E)** Western blot analysis was performed to assess the expression of apoptosis-related proteins. Data presented are the mean ± SD (*n* = 3). *p < 0.05, **p < 0.01, ***p < 0.001.

To determine the optimal dosage of H_2_O_2_, 661W cells were cultivated with 0, 25, 50, 100, or 200 μM H_2_O_2_, and the cell viability was measured by using a CCK-8 kit. Compared with the control group, cell viability was dramatically decreased by incubating with 50, 100 and 200 μM H_2_O_2_, while no significant decrease of cell viability was observed in cells treated with 25 μM H_2_O_2_ (Figure 5A). Thereafter, we determined the optimum time period of cell culture. We used different concentrations of Pae (0, 25, 50, 100 and 200 μM) to stimulate cells for 24, 48, or 72 h, and accessed cell viability to determine the adequate time of experiment. As shown in Figure 5B, we selected 48 h as the cultivation time to detect changes in the viability of cells treated with different concentrations of Pae. Thus, we measured the cell viability employing 100 μM H_2_O_2_ with different concentrations of Pae (0, 25, 50, 100 and 200 μM) treatment to sort out the moderate amounts of Pae. After 48 h *in vitro* culture, the cell viability was notably increased by 50–100 μM Pae and decreased by 200 μM Pae compared with H_2_O_2_ stimulation (Figure 5C). The results indicated that cell viability impaired by H_2_O_2_ could be effectively improved by Pae treatment in 661W cells.

Then, we examined the apoptosis-associated proteins including cleaved casepase-3, Bax and Bcl-2. The expression levels of pro-apoptotic cleaved caspase-3 and Bax were upregulated, whereas those of anti-apoptotic Bcl-2 were downregulated by H_2_O_2_ stimulation compared with the control group. These apoptosis-related alternations could be reversed by Pae treatment (Figures 5D,E). The results demonstrated that apoptosis induced by H_2_O_2_ could be significantly alleviated by Pae treatment in 661W cells.

### Paeonol Showed a Potential Antioxidative Effect in 661W Cells

**FIGURE 6:**
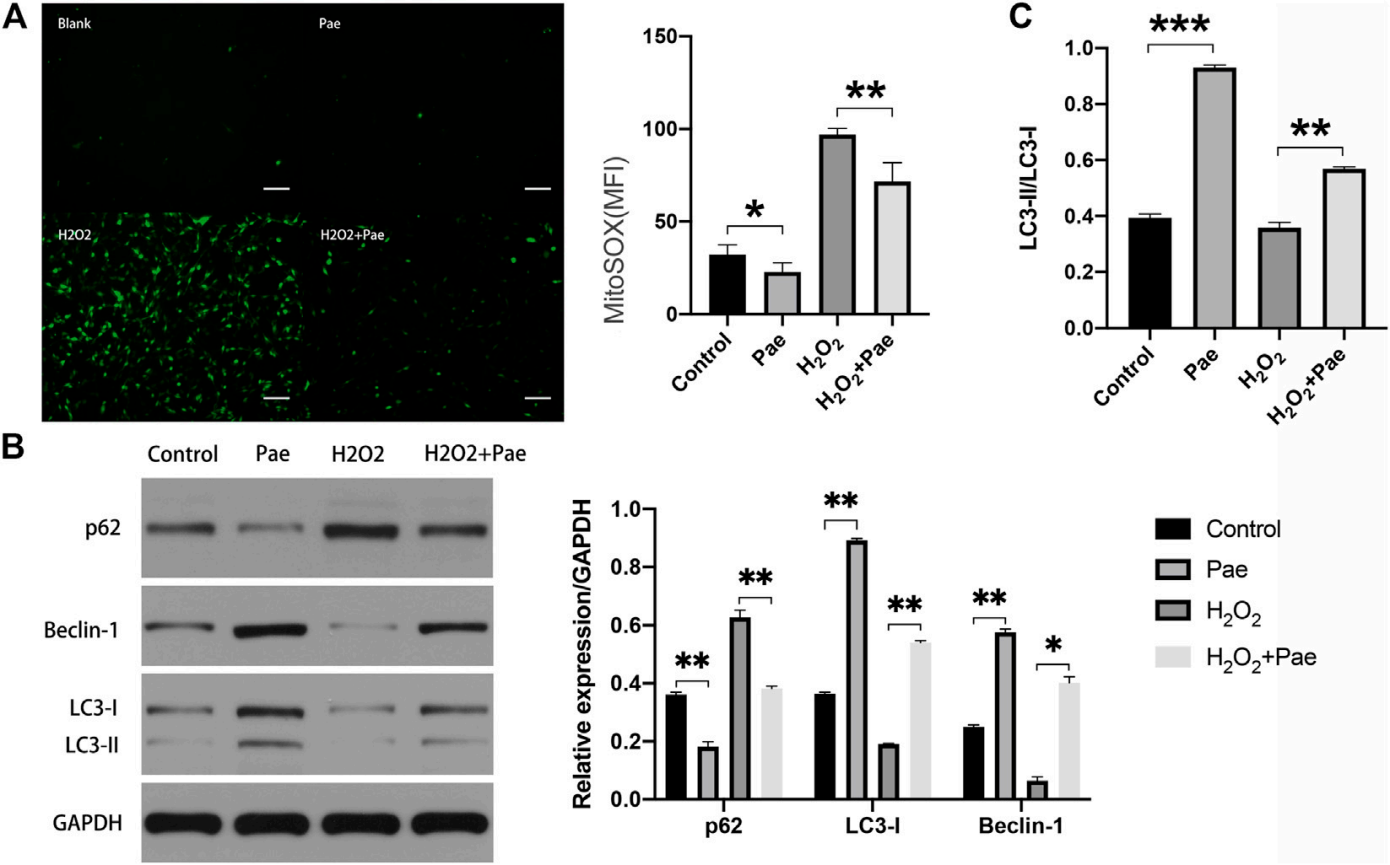
Impact of Pae on 661 W cells under H_2_O_2_ stimulation. **(A)** By using Mito-Sox assay, the mitochondrial ROS was determined to assess the levels of intracellular ROS of each group. **(B)** Western blot analysis showed the expression level of autophagy-related proteins. **(C)** The ratio of LC3-II to LC3-I was presented. The ratio was higher in Pae group comparing with in Control group and was higher in H_2_O_2_ + Pae group than H_2_O_2_ group. Data show mean ± SD. (*n* = 6–9 for each group) *p < 0.05, **p < 0.01, ***p < 0.001. Scale bar: 100 μm.

Subsequent experiments were conducted to determine whether Pae affected oxidative stress and autophagy in H_2_O_2_-stimulated 661W cells. By using Mito-Sox assay to detect the mitochondrial ROS, we found that the fluorescence intensity was significantly higher in the H_2_O_2_ group than the control group, and the fluorescence intensity noticeably decreased in the H_2_O_2_+Pae group, suggesting the antioxidative effect and protective effect of Pae on mitochondria in 661W cells (Figure 6A).

Furthermore, the expression of major autophagy marker proteins was measured (Figure 6B,C). After H_2_O_2_ stimulation, the expression levels of LC3-I, LC3-II and Beclin-1 were significantly downregulated, and the expression level of p62 were elevated compared with the control group (all *p* < 0.01). However, these changes of expression level could be reversed by the administration of Pae (all *p* < 0.01), which implied that Pae could activate autophagosome formation and enhance the autophagic flux in H_2_O_2_-stimulated 661 W cells. Significantly, the ratio of LC3-II to LC3-I was higher in Pae group comparing with in Control group and was higher in H_2_O_2_ + Pae group than H_2_O_2_ group. In combination with the expression of p62 and Beclin-1, it is possible that the autophagy overactivation was occur.

### Both the PINK1/Parkin Dependent and BNIP3L/Nix Dependent Autophagy Were Activated by Paeonol in 661W Cells

We speculated that Pae might regulate autophagy in 661W cells based on evidence obtained from above experiments. Thus, we measured the PINK1/Parkin dependent and BNIP3L/Nix dependent pathways of autophagy, respectively.

**FIGURE 7:**
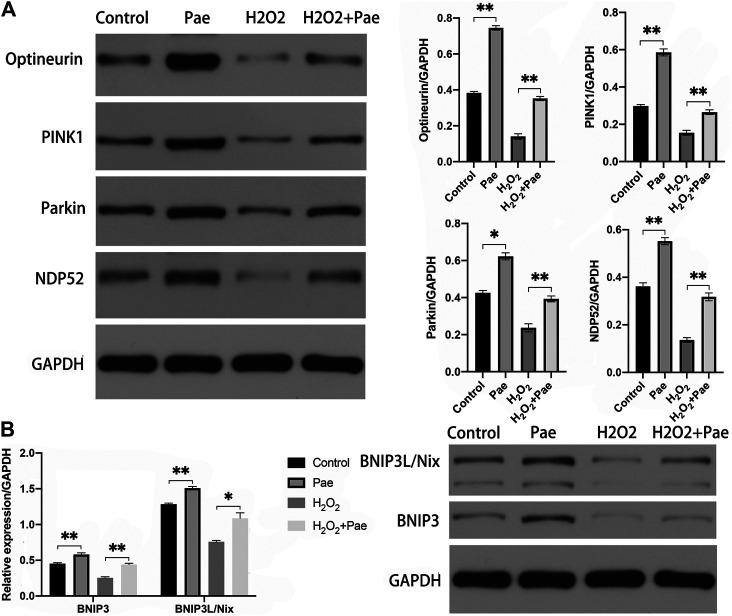
Pae activated mitophagy via the PINK1/Parkin dependent and the BNIP3L/Nix dependent pathways in 661W cells. **(A)** The expression of proteins associated with the classic INK1/Parkin dependent pathway of mitophagy was measured by Western blot analysis as well as **(B)** the expressed level of proteins of the BNIP3L/Nix dependent pathway of mitophagy. GAPDH, as loading control were reused from Figure.6. Data show mean ± SD. (*n* = 6–9 for each group) *p < 0.05, **p < 0.01.

The results of Western blot analysis showed that the expression of PINK1 and Parkin were significantly reduced after H_2_O_2_ stimulation (both *p* < 0.01), but were notably augmented after Pae treatment (both *p* < 0.01). It was worth noting that PINK1 and Parkin were dramatically upregulated by Pae treatment alone when compared with the control group (both *p* < 0.01) (Figure 7A). We further detected the expression levels of critical downstream proteins in PINK1/Parkin pathway and found H_2_O_2_-induced decreases of DNP52 and Optineurin were significantly reversed by Pae (both *p* < 0.01). In accordance with findings of PINK1 and Parkin, Pae treatment alone significantly augmented Optineurin (Figure 7A). The results showed that Pae could activated autophagy via the PINK1/Parkin pathway and might act on Optineurin directly.

In addition, we evaluated the BNIP3L/Nix dependent pathway of autophagy. Significantly, the expression levels of BNIP3 and BNIP3L/Nix were both decreased in the H_2_O_2_ group (both *p* < 0.01) but increased after Pae treatment (both *p* < 0.01) (Figure 7B), which indicated that Pae could activate autophagy via BNIP3L/Nix dependent pathway of autophagy in 661W cells as well.

## Discussion

As light-sensitive neurons, RPs are essential to the formation of vision, and the death and dysfunction of RPs could lead to irreversible vision loss and even blindness. However, the efficacy of several current strategies to protect photosensitive neurons remains unsatisfactory. RPs were known to have the highest density of mitochondria in the outer retina (Salminen et al., 2013). Mitochondria play an important role both in physiological functions including cellular metabolism, cell survival, and intracellular homeostasis and in various pathological conditions. Thus, we aimed to explore whether Paeonol, an anti-oxidant drug, could protect RPs from light-induced and H_2_O_2_-induced oxidative stress by regulating apoptosis and autophagy.

In this study, we found that Pae protects photoreceptors from oxidative stress-induced damage and preserved the number and function of photoreceptors. Consistent with previous studies (Russo et al., 2011; D'Adamo et al., 2020), the thickness of the ONL was reduced and the number of surviving RPs was also decreased in rats after LD stimulation. It could be assumed that Pae maintained the thickness of the ONL by increasing the number of RPs. Moreover, the results of TUNEL staining demonstrated that the percentage of apoptotic cells was decreased after Pae treatment and the results of TEM indicated that Pae-treated RPs had healthier mitochondria and greater number of mitophagosomes LD-induced RPs. The amplitudes of a-wave and b-wave are well correlated with the thickness of ONL and provide a direct and objective assessment of the variation in RP function (Jiang and Steinle, 2010; Scholz et al., 2015). Pae showed a protective effect on preserving the function of RPs function, with the reduced amplitudes of a- and b-waves in LD eyes being ameliorating after the administration of Pae.

We further explored the potential mechanism of the protective effects. Oxidative stress is a key factor in the secondary pathological process of outer retinal diseases. Considering that ROS accumulation can result in oxidative stress, which is mainly produced by H_2_O_2_ diffusion via aquaporins through biological membranes (Tan et al., 2004), we induced oxidative stress in cells using H_2_O_2_.

Oxidative stress-induced apoptosis leads to further injury and dysfunction of photosensitive neurons in outer retinal diseases. As an RP cell line that is sensitive to oxidative stress, the apoptosis of 661W cells can be stimulated by H_2_O_2_ (Weymouth and Vingrys, 2008). Thus, we used 661W cells to demonstrate the effects of Pae *in vitro*. The results showed that Pae can mitigate cellular apoptosis and impaired viability owing to H_2_O_2_ stimulation. In current study, the expression levels of Bax and cleaved casepase-3 were elevated, whereas the expression levels of Bcl-2 were downregulated after H_2_O_2_ stimulation, which were consistent with the pattern of Bcl-2-regulated pathway of mitochondria (Klionsky et al., 2016). Pae mitigated the expression of these proteins, showing that Pae decreased apoptosis by restraining the intrinsic apoptosis pathway in 661W cells. In contrast to our study, one earlier study considered Pae as a dose-dependent cell apoptosis inducer in tumor (Stone et al., 2008). Therefore, Pae should be employed carefully in present and further relevant researches are required.

Mitochondrial damage is strongly associated with oxidative stress (Kim et al., 2020). In order to investigate whether the damage was related to mitochondrial function, we deteced ROS levels, which was mainly produced in the mitochondrial electron transport chain with state III to state IV transformations in the organism. In the present study, ROS was significantly increased under H_2_O_2_ stimulation and largely alleviated by Pae treatment *in vitro*.

Furthermore, we found that autophagy can be stimulated by Pae through the PINK1/Parkin dependent pathway and the BNIP3L/Nix dependent pathway in 661W cells. Various pathways have been identified for different autophagy environments, such as the FUNDC1 pathway, the BNIP3L/Nix pathway, and the PINK1/Parkin pathway. The models of selective autophagy dictate the receptors, including major ones Optineurin and NDP52, link cargo to autophagosomal membranes, ubiquitination is recognized by these selective transporters. In our study, the expression levels of PINK1 and Parkin were upregulated, which could in turn trigger the overexpression of downstream proteins. As expected, SQSTM1/P62, Optineurin, and NDP52 were all downregulated under H_2_O_2_-induced injury and upregulated by Pae treatment, indicating that autophagy was increased in non-hypoxia-induced pathway in 661W cells. In addition, the expression levels of FUNDC1, BNIP3, and BNIP3L/Nix in different groups were consistent with those of PINK1/Parkin pathway associated proteins, revealing that it also plays an important role in this process. Our results showed that the conversion rate from LC3-I to LC3-II and the expression of Beclin-1 was repressed after H_2_O_2_ stimulation, along with overexpression of p62, revealing the resistance of autophagosome formation and the decreased autophagic flux caused by oxidative stress, which could all be effectively mitigated by Pae treatment.

This study still has limitations. The exploration of the relationship between Pae and autophagy is only in the preliminary stage, which requires more sophisticated experiments like the subcellular location and post-translational modification of PINK1 and Parkin matter, the immunofluorescence image and the gene-knockdown. The mutual relation between PINK1/Parkin dependent pathway and BNIP3L/Nix dependent pathway will also be investigated.

## Conclusion

This study showed that Pae exerted a protective effect on RPs via PINK1/Parkin dependent and BNIP3L/Nix dependent pathways of autophagy. More specific mechanism of mitochondrial autophagy is still need further study. Retinal diseases, especially which involved in retinal photoreceptor cells, is closely relevant to the processes of autophagy. It is feasible to further explore the mechanism of autophagy in retinal diseases for the prevention and treatment of retinal diseases. We will further study the role of Pae and the mechanism of autophagy, which may provide potential targets and new treatment schemes for retinal and mitochondrial related diseases.

## Data Availability

The original contributions presented in the study are included in the article/Supplementary Material, further inquiries can be directed to the corresponding authors.
